# “*We just don’t have the resources*”: Supervisor perspectives on introducing workplace-based assessments into medical specialist training in South Africa

**DOI:** 10.1186/s12909-023-04840-x

**Published:** 2023-11-06

**Authors:** Tasleem Ras, Louis Stander Jenkins, Colin Lazarus, Jacques Janse van Rensburg, Richard Cooke, Flavia Senkubuge, Abegail N Dlova, Veena Singaram, Emma Daitz, Eric Buch, Lionel Green-Thompson, Vanessa Burch

**Affiliations:** 1 University of Cape Town, Cape Town, South Africa; 2 Stellenbosch University and University of Cape Town, Cape Town, South Africa; 3 Walter Sisulu University, Mthatha, South Africa; 4 University of the Free State, Bloemfontein, South Africa; 5 Witwatersrand University, Johannesburg, South Africa; 6 University of Pretoria, Pretoria, South Africa; 7 Sefakho Makgatho University, Rankuwa, South Africa; 8 University of KwaZulu Natal, Durban, South Africa; 9 Colleges of Medicine of South Africa, Johannesburg, South Africa; 10 University of Cape Town & South African Committee Of Medical Deans, Cape Town, South Africa

**Keywords:** Specialist medical education, Workplace-based assessment, Resource-constrained setting, Barriers and enablers, Learning environment, Feedback, Entrustable professional activities

## Abstract

**Background:**

South Africa (SA) is on the brink of implementing workplace-based assessments (WBA) in all medical specialist training programmes in the country. Despite the fact that competency-based medical education (CBME) has been in place for about two decades, WBA offers new and interesting challenges. The literature indicates that WBA has resource, regulatory, educational and social complexities. Implementing WBA would therefore require a careful approach to this complex challenge. To date, insufficient exploration of WBA practices, experiences, perceptions, and aspirations in healthcare have been undertaken in South Africa or Africa. The aim of this study was to identify factors that could impact WBA implementation from the perspectives of medical specialist educators. The outcomes being reported are themes derived from reported potential barriers and enablers to WBA implementation in the SA context.

**Methods:**

This paper reports on the qualitative data generated from a mixed methods study that employed a parallel convergent design, utilising a self-administered online questionnaire to collect data from participants. Data was analysed thematically and inductively.

**Results:**

The themes that emerged were: Structural readiness for WBA; staff capacity to implement WBA; quality assurance; and the social dynamics of WBA.

**Conclusions:**

Participants demonstrated impressive levels of insight into their respective working environments, producing an extensive list of barriers and enablers. Despite significant structural and social barriers, this cohort perceives the impending implementation of WBA to be a positive development in registrar training in South Africa. We make recommendations for future research, and to the medical specialist educational leaders in SA.

**Supplementary Information:**

The online version contains supplementary material available at 10.1186/s12909-023-04840-x.

## Background

There have been some significant international shifts in the education of medical specialists over the past few years [1, 2]. These include the adoption of competency based medical education (CBME), increasing utilisation of workplace-based assessment (WBA), and the incorporation of WBA into systems of programmatic assessment in the context of CBME. For registrars (medical specialists‐in training), most of the educational contact between registrar and consultant (supervising medical specialist) happens ‘at the bedside’, and very little in the classroom. Continuous assessment of workplace performance following an iterative standardized in‐service process has the potential to bring assessment of clinical competence from an artificial context into the real world of clinical medicine, without compromising patient safety [3]. In South Africa (SA), the intended incorporation of WBA into medical specialist training programmes necessitated a review of current knowledge, practice and perceptions of WBA among those who would be implementing it.

WBA involves the assessor observing the trainee’s performance in the real world of clinical practice, providing feedback and ‘thus fostering reflective practice’ [4]. It ‘encompasses a wide range of assessment strategies’ [4] that collect and record information about trainees’ performance in the clinical setting. This information is then used to provide developmental feedback in formative assessments and make judgements in summative assessments. WBA is regarded as a valid and reliable means of assessment in health sciences education [5–7]. The reliability of WBA is established through adequate sampling, meaning that multiple encounters need to be observed by the assessor to achieve reliability [8, 9]. Since human observation and interpretation is a central feature of WBA the ‘assessment literacy’ [10] of the assessor—which includes knowing ‘what to look for, how to interpret, where to draw the line between satisfactory and unsatisfactory performance’ [1] —is critical. Robust and reliable decisions are reached by collating and evaluating sufficient information (data points) over a variety of assessment episodes, using information garnered along the way about trainee strengths and weaknesses to guide learning before a final decision is made [11]. International (mostly global north) experience has shown that when WBA is effectively implemented, assessment of competency is enhanced, and less emphasis is placed on the role of high stakes exit assessments, with all the variables that accompany this type of examination [11, 12]. WBA facilitates trainee learning by aligning real-world clinical experience, training program content, expected competencies, and assessment methods, providing feedback during or after observations, and using formative assessments to guide trainee learning towards desired outcomes [5]. As such, it is a form of ‘assessment *for* learning’ [13, 14]. Feedback and instruction become intertwined as the process of feedback does more than just report on student correctness or error but becomes the site of further guidance and instruction [15]. Hattie reports that the average effect size for feedback in school-level education, based on 12 meta-analyses, was 0.79 (twice the average) [15]. In a SA medical education context, Burch et al [16] reported that bedside feedback increased registrars’ confidence to undertake blinded patient encounters *without* consulting patient records prior to interviewing and examining the patient, with most students in the study recognising the learning value of feedback in terms of information-sharing, motivation, and learning behaviour. Veloski et al’s [17] systematic review demonstrated an overwhelmingly positive impact of feedback on clinician performance, being significantly impacted by the source of feedback and its duration.

A comprehensive WBA framework takes place in a socially situated space (the health facility), with clearly defined actions (well defined learning outcomes, standardised workplace formative assessments and feedback) and actors with specific roles to play (capacitated, engaged staff and students) [5, 18]. Its implementation is deeply influenced by the context in which it is practised, being grounded in the social realities of the workplace [19, 20]. Given the centrality of feedback to the process of WBA, it becomes apparent that the institutional culture and relationship between supervisors and registrars are key factors that influence the assessment outcomes [21, 22]. Student engagement in the process of WBA is also integral to its success, and attention should be paid to the social nature of learning [18]. Medical education and learning are embedded in, and shaped by, the social context in which they take place [23] and the power relations between consultant and registrar [24]. Becoming a doctor, as Lave and Wenger argue in their social learning theory, entails making the socially situated journey from being a ‘legitimate peripheral participant’ to being a fully-fledged member of a ‘community of practice’ [25, 26] thus acquiring the identity shared by other members of the community [23] and becoming a new kind of person. The socio-cultural dynamic, whether it is contextual or interpersonal, must be understood if WBA is to be an effective educational approach.

In SA, the social space and the interpersonal interactions are vulnerable to dysfunction [27], as demonstrated by the South African student movements in the recent past and one cannot assume that the relationships in clinical and educational spaces are functional and healthy [28–30]. Issues of discrimination, barriers related to racism, sexism, and favouritism were also found to have negative impact on the specialist training programs in South Africa [31]. Any attempt at implementing WBA in this context would need to take this reality into consideration [32]. Given the impetus needed to change workplace practice and develop assessment literacy, a significant demand on resources is made. Recent work in a postgraduate training program in South Africa highlighted the multiple demands for training and supervision resources needed [33]. In the context of developed countries, the development and implementation of WBA strategies were resource intensive [34, 35], and given the realities of lower-middle income countries (LMIC), a local response based on local realities is needed.

The impetus for implementing WBA in SA medical specialist training programmes is growing. The Colleges of Medicine of South Africa (CMSA), as the examining body for medical specialists in South Africa, has called for the integration of WBA as a core practice in training programmes [36]. This call is supported by the SA Committee of Medical Deans (SACOMD), representative of all health science faculties in the country. This collective intent to incorporate WBA into the SA context raises an important research question. Given the resource challenges of implementing a comprehensive WBA framework, the paucity of data on WBA in LMICs, and the social complexity alluded to above, what is the state of readiness of training programmes in SA to implement WBA? To answer this question, a rapid situational analysis was performed, aimed at generating data reflective of local SA realities. The key outcomes reported are a quantification of current knowledge and practices, and qualitative perceptions of programme managers and clinical supervisors of potential barriers or enablers to the successful implementation of WBA. We report on the latter, qualitative, outcome in this paper.

## Methods

This was a cross-sectional, mixed methods observational study using a parallel convergent design. This is an appropriate design for this type of study as we collected the two types of data simultaneously, with the intention that they would converge post-analysis to provide a comprehensive overview of the phenomenon being studied and inform WBA design and implementation strategies [37]. In the instance of this study, the quantitative component provided data as measured against a set of objective questions based on the literature, while the qualitative component allowed participants to express their perceptions based on their subjective interpretations of their own experiences and knowledge.

The setting encompassed all South African universities offering medical specialist training programmes. This included nine health sciences faculties, spanning all medical specialties and sub-specialties. The official language for this training is English. All training sites are accredited by the Health Professions Council of South Africa (HPCSA) and funded by the National Department of Health of South Africa. It should be noted that WBA has not yet been formally adopted at any of these training sites, so participants’ knowledge and exposure to WBA praxis is reflective of the pre-implementation phase that the country is in.

Participants were drawn from all participating institutions (nine health sciences faculties in South Africa), using a purposive and snowball sampling method. The inclusion criteria were: currently a clinical supervisor of registrars OR manager of a registrar-training programme. This means that all participants were medical doctors with specialist registration, as this is a requirement of these positions. We did not stipulate a minimum or maximum time employed in the current position. There were no exclusion criteria applied. Participants were recruited by collaborators from their own institution, either directly by telephone or email, or via institutional mailing lists. We estimated that a sample of two hundred and sixteen (N = 216) respondents would represent about 80% of the training programmes in SA, which would constitute an acceptable representation of this population.

Data was collected using a self-administered online questionnaire (Appendix A). This novel questionnaire was developed by the research team using literature cited above to identify key knowledge and practice elements of WBA. Two open-ended questions were also included. It was scrutinised individually by a panel of medical educational researchers and practitioners who provided email feedback on content and face validity. After these comments were incorporated, the research team met and reached consensus on the finalised tool. Minor changes were incorporated at this stage: two questions on WBA practice were added; and items were separated into knowledge and practice domains. The questionnaire was then loaded onto the Google online platform as a fillable form, with the introductory section being the informed consent component. This online version was piloted with nine participants who were not part of the study sample. There were no changes made to the tool after this pilot study.

Responses from the completed questionnaire were automatically uploaded to a Google Sheet and downloaded as a Microsoft Excel spreadsheet. The qualitative data was extracted from this spreadsheet for analysis manually. Using Braune and Clarke’s (2006) six-step guide to thematic analysis [38] and an inductive approach, the qualitative data was iteratively read with the questions in mind, after which a set of codes were generated by a member of the research team (ED) trained in qualitative research. The codes were categorised according to their content, and where these categories were coherent, themes emerged. The themes were discussed with the lead author (TR), who also has qualitative research experience, who reviewed the analysis process to ensure that the trustworthiness criteria as defined by Lincoln and Guba were met [39].

## Results

**Fig. 1:**
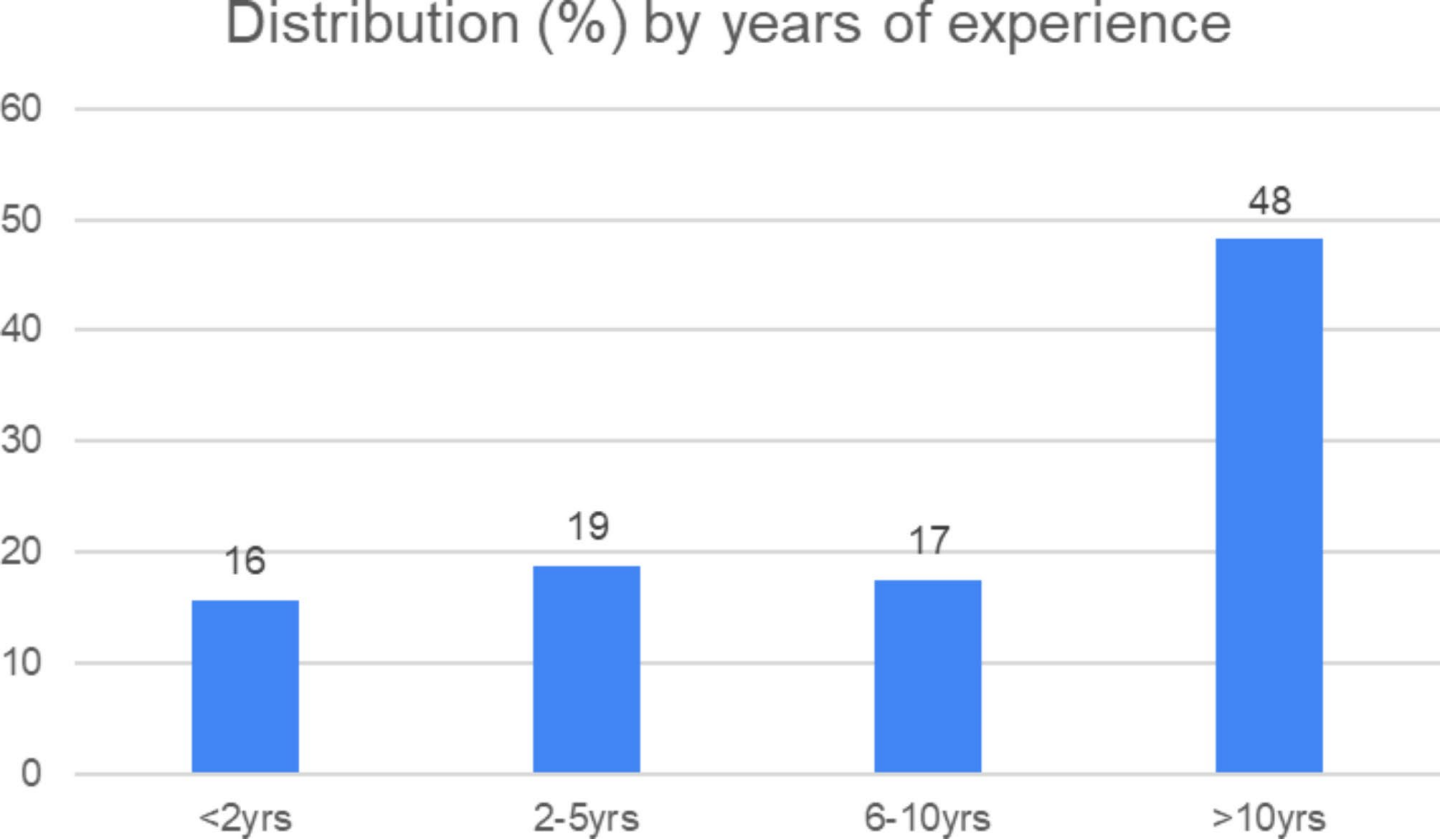
Distribution of respondents by years of experience in medical specialist training

A total of one hundred and sixty-six (n = 166) individuals representing forty-four different specialty or sub-speciality training programmes and nine health sciences faculties in SA completed the online questionnaire. This represents 76% of the intended sample size (N = 216). Eighty-five respondents (51.2%) of the sample self-identified as programme convenors/managers, with the balance self-identifying as supervisors. Figure 1 indicates the relative experience within these roles.

Participants were asked to respond in text to two open-ended questions: “What are your experiences/perceptions of factors in your clinical/academic environment that are/will be *barriers* to the success of WBA?” and “What are your experiences/perceptions of factors in your clinical/academic environment that are/will be *enablers* to the success of WBA?”. These responses yielded four themes. These themes were ‘Structural readiness and support’, ‘Staff capacity’, ‘Quality assurance’ and ‘Social dynamics of WBA’.

### Structural readiness and support

The structural issues identified for implementing WBA raised by respondents were related to time, staff shortages, equipment deficiencies, perceived lack of stakeholder buy-in and the technology needed for WBA implementation.

Time needed for workplace training when weighed up against the clinical demands and perceived staff shortages emerged as a central concern.

“The biggest problem is time. In an environment with limited lecturer/student ratio pre-and post-graduate, it is impossible. The clinical demand on consultants/supervisors is preventing intense WBA on a daily basis.” (R138).

Respondents related this perceived lack of time to staff shortages leading to exigent clinical workloads and unfavorable ratios between consultants and registrars:

“Correct, adequate and accurate WBA need trained staff to be available and involved. Staff are currently overwhelmed with workload. Adding what I would feel is appropriate and fair WBA in all aspects—operating, patient assessment, patient presentations, theory etc. will need more resources—specifically more staff.” (R3).

Many respondents felt that there simply weren’t enough supervisors available to train and assess registrars as per the perceived demands of WBA in the current environment:

“…aligning availability of supervisors and registrars… not enough supervisors for number of assessments needed.” (R44).

In small disciplines and departments, this shortage of supervisors would be exacerbated by the perceived demands of WBA:

“Small disciplines and departments, where there could be one consultant, and service delivery issues.” (R43).

However, despite the heavy workload and shortages, respondents identified enabling opportunities within these spaces. The busy clinical workplace offers opportunities for exposure to a wide range of patients and clinical encounters that constitute the basis of WBA.

“Clinical service delivery areas that are busy will offer [an] opportunity for registrar exposure [to] different conditions…” (R113).

“Sufficient work-based opportunities exist that can provide assessment opportunities if there is cohesion, clarity and communication.” (R72).

Respondents identified a need for all stakeholders to buy into WBA and for proper institutional support to make it work. Resistance or lack of support from supervisors was identified as a potential stumbling block:

“Not all supervisors buy into the process. Some still have very archaic ideas how to evaluate and support (not support) (sic.) registrars and this is very difficult to change.” (R110).

The need for institutional buy-in and support was also highlighted:

“Buy-in from the Department of the reliability of such assessments.” (R136).

“We will need practical support from our University—which is lacking quite often.” (R55).

They had clear ideas of the structural and institutional enablers required for WBA, though some of them are aspirational. Three key groups were identified whose support and buy-in to the implementation of WBA they perceived as critical: institutions and administrators, consultants, and registrars. They described the need for leadership coming from Deans and Heads of Department (HoDs) and the authoritative structures to support the implementation of WBA:

“…HoD buy-in, lots of energy on change management.” (R12).

“The support of the Deanery in appreciating the value of WBAs.” (R80).

“Provincial (government) buy-in mandating the assessments and providing the opportunities on the clinical platform.” (R86).

One respondent suggested a “A single national co-ordinated process led by each CMSA college.” (R9) This is an important stakeholder as the CMSA (Colleges of Medicine of SA) is the sole examining authority for medical specialists in the country.

Respondents identified a well-regulated environment as being an enabler of the successful implementation of WBA. The need for clear guidelines, regulation, and monitoring was identified.

“Clear guidelines from the CMSA, SACOMD and Universities. Making it a mandatory requirement for progression in training.” (R4).

The importance of registrar and consultant support and buy-in was also noted, with respondents noting perceived buy-in from registrars, as shown by the following excerpts.

“Registrars thus far have really appreciated the feedback so there is a huge “buy-in” from trainees’ side.” (R62).

“The willingness by the trainers to learn and train others is a positive factor.” (R122).

The final structural issue identified by respondents identified a lack of and need for adequate technological support to capture, store, and manage assessments.

“Then, another barrier—too often we are asked to do everything ourselves—develop the assessments, do them and submit them—this needs a good electronic system that is outsourced and well managed, so that reminders are sent, data is stored appropriately and technical issues are sorted by the team as opposed to the assessors struggling to submit the assessments.” (R3).

Again, as with other barriers, the respondents offered enabling factors that address the technology concern. Existing technologies in the WBA space were seen as enabling the implementation of WBA.

“The groundwork and experiences using (*a commercial software programme*) and (*another commercial software programme*) has already been laid and the supervisors already have experience with this.” (R10).

“An already existing e-portfolio and WBA.” (R49).

### Staff capacity

Supervisor capacity was seen as central to WBA implementation. Many respondents simply stipulated that training of trainers/supervisors was lacking as well as essential for an alternative system of assessment to work.

“The training of supervisors to be able to accomplish this.” (R23).

“Lack of training of supervisors/trainers…” (R32).

Respondents cited the general lack of supervisor training, variability of assessment skills, knowledge, and experience of WBA among supervisors, and the need to monitor the competence of clinical teachers.

“Supervisors who may not be keen to participate in the WBA (due to lack of knowledge/skills) and lack of resources.” (R137).

“Adequate training of and continuous monitoring of WBA competence of clinical teacher.” (R114).

Significantly, only one respondent identified feedback as an element that would require training if it were not to become a barrier to successful WBA: “…supervisor training especially on feedback.” (R33).

While lack of training has been mentioned as a barrier, respondents reported positive attitudes to implementing WBA, as highlighted by the following quotations that demonstrate respondents’ commitment to ensuring that their registrars receive good training.

“The ability and willingness for pathologists in the unit to perform the WBAs on a monthly basis currently. The value of continuous WBAs are appreciated by both pathologists and registrars in preparing them for exit exams and professional practice. The support of the Deanery in appreciating the value of WBAs.” (R80).

“I think it will be welcomed by trainees as it will provide them with constant feedback on “how they are doing”. In principle both myself and co-supervisor are in agreement that WBA could be a very useful tool—so attitude is positive.” (R104).

### Quality assurance: subjectivity and standardisation

Respondents noted the role that subjectivity might play in the assessment process, making it potentially unfair to registrars:

“Very small numbers of both registrars and supervisors […] That makes it very difficult to remain objective as we work very closely with fellows and develop a personal relationship.” (R104).

Others noted time constraints as a barrier to objectivity in assessments:

“Assessments are also subjective (hence you need more which requires more time). An assessment that is expressed as a score gives false re-assurance compared to feedback only.” (R22).

Some worried that the narrow gap in seniority between registrars and junior consultants could impact the reliability of WBA.

“Junior consultants assessing registrars (whom they are barely senior to) too leniently, leading to a drop in standards.” (R24).

Respondents also expressed the perception that there was “inconsistency and subjectivity of assessments between disciplines” (R117) and that this would hinder the successful implementation of WBA. Some suggested that WBA would “need to have multiple assessors to achieve reliability and validity” (R10). This was echoed by another respondent who pointed out that:

“WBA is also a reflection of the teacher, so the teacher should not be doing the examination. It needs to be non-biased, or at least include an examiner that was not involved in teaching that section, preferably an external examiner” (R162).

Respondents also commented on the need to standardize the WBA process and its component parts. Three areas in need of standardization/agreement were identified: EPAs, Standard Operating Procedures (SOPs), and benchmarking.

A general lack of and need to standardize/agree on WBA and its components was identified by respondents, which may be a complex task given the variations across service platforms.

“The WBA would need to be simple and standardized—for this to occur, they need to be developed appropriately by a task team.” (R3).

“We need to agree on standards and expectations of the registrars in each division, this may be difficult.” (R5).

To achieve such standardization across training centers respondents identified the need to standardize the curriculum and EPAs:

“Agreement around curriculum/blueprint.” (R116).

“Agreeing on EPAs and the frequency and style of assessment.” (R9).

They also identified a lack of benchmarking and across different contexts, and the inconsistent use of clinical guidelines as a barrier:

“Benchmarking not standardized—supervisors having differing expectations….” (R39).

“Needs all the Universities to agree as well on certain practices/SOP’s/guidelines.” (R54).

Respondents further expressed concern about the lack of standardization between clinical exposure in certain disciplines, and between different clinical platforms, even possibly within the same discipline.

“Large registrar numbers in a department tend to rotate quickly through the disciplines and move on before they are fully competent in that discipline. The WBA can be unfair to some registrars for that reason.” (R29).

“…some facilities have inferior equipment and infrastructure even just simple stuff like internet access is a problem at [a named] tertiary hospital.” (R105).

“…not all training centres have equivalent facilities for training in specific areas of the subspecialty…” (R83).

### Social dynamics of WBA

Some respondents commented on interpersonal relationships between consultants and registrars as a barrier to successfully implementing WBA. Furthermore, some consultants were aware of the role bias and favoritism could play in making WBA successful or not.

“Some clinicians might not see potential in a registrar or may not like him/her personally and might act with bias.” (R19).

“Biases towards a particular registrar.” (R60).

“Lack of objectivity by supervisors. Favouritisms (*sic*).” (R118).

“Unrecognised bias (lack of self-awareness).” (R119).

“Personality clashes between supervisor and registrar may result in bias.” (R148).

The central importance of the quality of the relationship between consultants and registrars to WBA was also noted as potential barriers.

“Supervisors/trainees poor relationship.” (R52).

“Not all supervisors buy into the process. Some still have very archaic ideas how to evaluate and support (not support)(*sic*) registrars and this is very difficult to change.” (R110).

Alternatively, the closeness of the working relationship between supervisor-registrar was seen as a potential enabling factor, based on respondents own past experiences.

“We already spend a lot of one-on-one time with our registrars and WBAs can very easily be incorporated in these sessions in a structured way.” (R17).

“Already close one-on-one engagement between supervisors and registrars, which will facilitate assessment.” (R34).

## Discussion

In this paper we describe the qualitative findings from a mixed methods study in which we explored the perceptions of supervisors of postgraduate specialist trainees regarding barriers and enabling factors likely to impact upon the implementation of WBA in South Africa. Principally, the themes speak to the importance of context for the implementation of WBA. This context refers to the formal systems within which learning takes place, and the informal, cultural or interpersonal aspects of the training spaces.

Our findings indicate that there are substantive concerns about the lack of resources to implement WBA in the SA context. This finding is closely linked to the quality assurance theme, both representing aspects of WBA that requires interventions at policy, governance and leadership levels. Like previous studies [31], systemic barriers and enablers had to do with the resource constrained environment in which supervisors/trainers must operate. This included underfunding, understaffing, exigent clinical environments and caseloads—all of which led to a perceived lack of time to do the assessments that WBA requires—and inadequate infrastructure including a lack of equipment, diagnostic platforms, and limited access to reliable internet connectivity in the workplace. Appreciating these concerns against the backdrop of the cost of implementing WBA in well-resourced contexts [34, 35] provides further opportunity for reflection, especially around long-term sustainability of WBA in a LMIC. However, there is evidence from other resource-constrained environments such as Pakistan [40] that WBA can be adapted to resource-scarce contexts if the design and implementation process is sensitive to these contextual realities. This discussion emphasises the need for effective alignment and collaboration between decision-making structures, those with access to resources, and those tasked with implementing WBA in clinical spaces.

An interesting finding within the ‘staff capacity’ theme was the self-identified need for ongoing training and the seemingly high levels of motivation to adopt WBA among the respondents. This high level of motivation for change may represent a frustration with current practices, though this was not explored in this study. This level of motivation was replicated empirically in a SA study which documents a pilot WBA implementation in a general surgery training programme [31]. Respondents also perceived buy-in for WBA to be high both among supervisors and registrars, and despite the many challenges faced, all respondents stated a commitment to ongoing WBA practices. When viewed from the perspective of change management, a motivated and pro-active cohort, with a clear understanding of the task at hand, is critical to effective and sustainable implementation of new practices [41]. That leading structures such as the SACOMD and CMSA are fully in support of these efforts lends impetus to the high levels of motivation expressed amongst respondents. The convergence of intentional leadership and engaged, capacitated staff would bode well for the sustained implementation of WBA.

The supervisor-registrar relationship, mentioned by our respondents as the basis for the learning encounters in the workplace, must receive due consideration. Where these relationships are found to be dysfunctional, learning is materially impacted [27]. In line with existing literature [31] on the social factors that affect registrars in their training in South Africa, there was a recognition by respondents that the supervisor-registrar relationship is not always healthy or functional and may be characterised by bias, victimization, and favouritism. Proactively pursuing healthy relationships that affirm student competency as an educational imperative has been shown to enhance learning outcomes in postgraduate education [42]. With feedback being so central to clinical learning, as evidenced by multiple studies in this area [16, 17, 19, 20], and the supervisor-registrar relationship being the micro-platform for the delivery of effective feedback, the importance of functional relationships in WBA praxis is further enhanced. This praxis should therefore not only focus on modes of feedback, but should explicate the relationship as a platform for trustworthy engagement.

### Limitations

The key limitation of the study is that we did not canvas the opinion of registrars regarding the barriers to, and enabler of, WBA in South Africa. This requires a separate in-depth study that will provide critical information from the perspective of trainees.

This exploratory study provided a superficial sense of perceptions of supervisors to WBA implementation in a SA context. As such, deep inferences about the learning environment cannot be made from this dataset. Additionally, while a fairly good response rate was achieved, the perspectives of those supervisors who did not complete the survey is not known—these missing participants may conceal perceived barriers and enablers that were not uncovered in this study.

A third limitation is that we only collected data from respondents via the online questionnaire, expecting them to type their responses. This may have limited the depth of their contributions when compared to verbal reports, which could have produced more depth.

## Conclusion

We conducted an observational cross-sectional mixed methods study in a resource constrained context and report the qualitative data here. Supervisors and convenors demonstrated good insight into their respective working environments, producing an extensive list of barriers and enablers. Future research should focus on expanding the stakeholder engagement to include registrars, health facility managers and policy-makers, experiences of these stakeholders of early WBA implementation, explore novel WBA practices that respond to low-resourced contexts, and the social dimensions of WBA (including patient and community engagement).

We make the following recommendations to aid WBA implementation in South Africa:

1. An intentional alignment between all decision-making bodies during the design and early implementation phase, as well as a consensus-based monitoring process.
2. That the financial and non-financial costs pertaining to technology, staff capacity-building and ensuring that institutional regulations are adapted be made clear.
3. Social and interpersonal factors must be taken into consideration when initiating WBA practices within clinical spaces.
4. A standardised monitoring and evaluation process should be implemented to document the progress being made.
5. A structured pathway towards staff capacitation should be developed, funded and implemented.

### Electronic supplementary material

Below is the link to the electronic supplementary material.

Supplementary Material 1

## Data Availability

The datasets used and/or analysed during the current study are available from the corresponding author on reasonable request.
