# Supplementary material for: “We just don’t have the resources”: Supervisor perspectives on introducing workplace-based assessments into medical specialist training in South Africa
Source: BMC Med Educ. 2023 Nov 6;23:832. doi: 10.1186/s12909-023-04840-x (PMC10629100; doi:10.1186/s12909-023-04840-x)
Supplement: Supplementary file 1 — Supplementary Material 1 [file 12909_2023_4840_MOESM1_ESM.docx]

**What is your roleAppendix 1**

**Data collection tool** (*this tool will be converted to an online survey tool using RedCap or Survey Monkey)*

**Demographics/Biographics:**

1. Name of University

2. Name of MMed specialty

3. Role at university

4. Years involved with registrar training/medical education

| **Objective 1 : Describe broad knowledge of and attitude to WBA** | |
| --- | --- |
| 1. I am familiar with Workplace-based assessments. | - Agree - Disagree |
| 1. I heard about WBA from: | - Colleagues - Conferences - Journals - Public media - Other (specify) - Not yet |
| 1. WBA are valid and reliable methods of assessment for registrar training | - **agree** - disagree - Unsure |
| 1. WBA has value for both formative and summative assessments of registrar training | - **Agree** - **Disagree** - **Unsure** |
| 1. WBA requires specific training for supervisors to enhance validity and reliability | - **Agree** - Disagree - Unsure |
| 1. WBA is built on a framework of Entrustable professional Activities (EPAs) that are aligned with the curriculum | - **Agree** - **Disagree** - Unsure |
| 1. EPAs are activities that can be directly observed in the workplace | - Agree - Disagree - Unsure |
| 1. EPAs represent a quantified level of trust that supervisors have in registrars, based on the observed competence within a particular domain | - **Agree** - Disagree - Unsure |
| 1. An entrustment decision for an EPA can only be made once a registrar has reached a pre-defined level of competence within a particular domain | - **Agree** - **Disagree** - Unsure |
| 1. There are adequate resources for the implementation of WBA in my discipline/institution? | - **Agree** - **Disagree** - **Unsure** |
| 1. I support the implementation of WBA in the registrar training programme? | **• Agree**  **• Disagree**  **• Unsure** |
| 1. How often do you think WBA should be implemented? | - Daily - Weekly - Monthly - Quarterly   - Other: |

| **Objective 2: Describe broad practices of WBA (*please complete if you use any form of workplace assessments in your programme*)** |  |
| --- | --- |
| 1. We regularly use some of the tools of WBA for workplace evaluations to monitor registrar progress (e.g. mini-CEX, DOPS, multi-source feedback) | **weekly**  **monthly**  **quarterly**  **annually**  **never** |
| 1. Our supervisors are trained to ensure accurate assessment of workplace performance | - **Agree** - **Disagree** - **Unsure** |
| 1. We have a standardised format of recording workplace assessments | - **Agree** - **Disagree** - **Unsure** |
| 1. Our WBAs include an emphasis on direct observations by various supervisors in different clinical settings. | - **Agree** - **Disagree** - **Unsure** |
| 1. Our supervisors give regular feedback to registrars, who reflect on this in learning conversations with their supervisors. | - **Agree** - **Disagree** - **Unsure** |
| 1. We have structured, agreed upon methods to score registrars’ level of competence | - **Agree** - **Disagree** - **Unsure** |
| 1. We have regular supervisory meetings to discuss registrar progression | - **Agree** - **Disagree** - **Unsure** |
| 1. We use these workplace evaluations to make decisions about registrars’ competence and/or progression | - **Agree** - **Disagree** - **Unsure** |
| 1. We only use internal examinations to make decisions about registrar progression | - **Agree** - **Disagree** - **Unsure** |
| 1. Our College is pursuing WBA as an assessment method of the future, as part of programmatic assessment | - **Agree** - **Disagree** - **Unsure** |
| 1. Our university’s regulations cater for WBA as a formal assessment method | - **Agree** - **Disagree** - **Unsure** |
| 1. Our supervisors are able to deliver on the expectations of WBA | - **Agree** - **Disagree** - **Unsure** |
| 1. If you use a standardised format of recording the WBA activities, please indicate the format in which it is recorded | - **Paper** - **Digital** - **Combined** - **Other (specify)** |

| **Objective: Describe (perceived) barriers/risks or enablers/opportunities to/for WBA – *open-ended*** | |
| --- | --- |
| 1. What are your experiences/perceptions of factors in your clinical/academic environment that are/will be barriers to the success of WBA | *Free text answer* |
| 1. What are your experiences/perceptions of factors in your clinical/academic environment that are/will be enablers/opportunities to WBA | *Free text answer* |
